# Possible mechanisms of SARS-CoV-2-associated myocardial fibrosis: reflections in the post-pandemic era

**DOI:** 10.3389/fmicb.2024.1470953

**Published:** 2024-10-08

**Authors:** Zhan Wang, Luwei Li, Shuai Yang, Zhengrui Li, Pengpeng Zhang, Run Shi, Xing Zhou, Xiaojuan Tang, Qi Li

**Affiliations:** ^1^Department of Urology, The First Affiliated Hospital of Zhengzhou University, Zhengzhou, China; ^2^Department of Pediatric Neurology, The First Affiliated Hospital of Zhengzhou University, Zhengzhou, China; ^3^The Third Clinical Medical College of Zhengzhou University, Zhengzhou, China; ^4^Department of Oral and Maxillofacial-Head and Neck Oncology, Shanghai Ninth People’s Hospital, Shanghai Jiao Tong University School of Medicine, Shanghai, China; ^5^Department of Lung Cancer, Tianjin Lung Cancer Center, National Clinical Research Center for Cancer, Key Laboratory of Cancer Prevention and Therapy, Tianjin's Clinical Research Center for Cancer, Tianjin Medical University Cancer Institute and Hospital, Tianjin, China; ^6^Department of Oncology, The First Affiliated Hospital of Nanjing Medical University, Nanjing, China; ^7^Department of Pediatric Surgery, The First Affiliated Hospital of Zhengzhou University, Zhengzhou, China; ^8^Department of Plastic and Reconstructive Surgery, The First Affiliated Hospital of Zhengzhou University, Zhengzhou, China

**Keywords:** COVID-19, myocardial fibrosis, TGF-β1, RAAS, mechanisms

## Abstract

Since December 2019, coronavirus disease 2019 (COVID-19) has been spreading worldwide with devastating immediate or long-term effects on people’s health. Although the lungs are the primary organ affected by COVID-19, individuals infected with SARS-CoV-2 also develop systemic lesions involving multiple organs throughout the body, such as the cardiovascular system. Emerging evidence reveals that COVID-19 could generate myocardial fibrosis, termed “COVID-19-associated myocardial fibrosis.” It can result from the activation of fibroblasts via the renin-angiotensin-aldosterone system (RAAS), transforming growth factor-β1 (TGF-β1), microRNAs, and other pathways, and can also occur in other cellular interactions with SARS-CoV-2, such as immunocytes, endothelial cells. Nonetheless, to gain a more profound insight into the natural progression of COVID-19-related myocardial fibrosis, additional investigations are necessary. This review delves into the underlying mechanisms contributing to COVID-19-associated myocardial fibrosis while also examining the antifibrotic potential of current COVID-19 treatments, thereby offering guidance for future clinical trials of these medications. Ultimately, we propose future research directions for COVID-19-associated myocardial fibrosis in the post-COVID-19 era, such as artificial intelligence (AI) telemedicine. We also recommend that relevant tests be added to the follow-up of COVID-19 patients to detect myocardial fibrosis promptly.

## Highlights

- SARS-CoV-2 activates cardiac fibroblasts through pathways including RAAS, TGF-β1, microRNAs, Glycolysis, Mitochondrial metabolism, and DAMP.
- In the RAAS system, factors such as Ang II and AT1R promote fibrosis, whereas Ang 1–7, Ang 1–9, and AT2R have inhibitory effects. In addition, ACE2 may play a dual role.
- TGF-β1 has not only pro-fibrotic but also immunosuppressive effects. It could be a potential predictor of prognosis in COVID-19 patients.
- SARS-CoV-2 infection promotes macrophage recruitment and a shift to a pro-fibrotic phenotype.
- The synthetic glucose analog 2-DG has been subjected to Phase II clinical trials and the results indicate that 2-DG is effective in treating COVID-19.

## 1 Introduction

COVID-19 has been popular since the end of 2019 and the pandemic is still wreaking havoc, placing an alarming burden on the health of people around the world. Severe acute respiratory syndrome coronavirus 2 (SARS-CoV-2), the pathogenic agent of COVID-19 (Zhou et al., 2020), can cause complications in multiple systems throughout the body other than the respiratory system, such as the circulatory system (Zheng et al., 2020; Chung et al., 2021; Tobler et al., 2022), the reproductive system (Sheikhzadeh Hesari et al., 2021), the digestive system (Cao et al., 2021), the nervous system (Chen et al., 2022), and the endocrine system (Szczerbinski et al., 2023). For example, SARS-CoV-2 infects host cells via ACE2 receptors, leading to acute myocardial injury and chronic damage to the cardiovascular system (Zheng et al., 2020). Evidence of myocardial injury has been reported in more than 60% of patients hospitalized for COVID-19 (Giustino et al., 2020), approximately 10% developed palpitations after discharge, and 5% had persistent chest pain months later (Huang C. et al., 2023; Lima and Bluemke, 2021). Thus, in addition to short-term injury, many patients still present with a range of symptoms after the elimination of acute infection (Xiong et al., 2021; Lu et al., 2023).

Myocardial fibrosis is a progressive pathological process elicited by the activation and excessive proliferation of myocardial fibroblasts and the deposition of extracellular matrix (ECM) (Lopez et al., 2021). It involves cardiac fibroblasts, cardiomyocytes, immune cells, and other cells, and it occurs under the action of a variety of pro-fibrosis factors, such as TGF-β1. There is a complex relationship between these factors depending on and restricting each other. However, the factors for myocardial fibrosis caused by cardiovascular diseases differ (Lopez et al., 2021), in which viral infections occupy an important place (Rafiee and Friedrich, 2024; Kenney et al., 2022).

An increasing number of studies have confirmed the potential correlation between COVID-19 and myocardial fibrosis. Cardiovascular complications are common in severe infections and have been reported in sepsis and a variety of viral diseases, such as COVID-19 and yellow fever (Chung et al., 2021; Giugni et al., 2023; Wang et al., 2020; Habimana et al., 2020). In addition, during the acute phase of SARS-CoV-2 infection leukocytes may secrete large amounts of inflammatory chemokines and cytokines (IL-1, IL-4, IL-6, IL-10, and CCL2, etc.), accelerating fibrosis (Espin et al., 2023; Del Valle et al., 2020; Chan et al., 2023). Myocardial fibrosis may potentially arise from acute phase myocarditis and myocardial injury, given that instances of myocardial fibrosis have been documented in patients suffering from COVID-19 several months following their hospitalization (Parhizgar et al., 2023; Kounis et al., 2024). Several studies have reported that cardiac magnetic resonance imaging in COVID-19 and/or post-COVID-19 patients showed late gadolinium enhancement (LGE) suggestive of myocardial fibrosis, particularly those experiencing a severe COVID-19 course (Morrow et al., 2022; Raafs et al., 2022; Bakhshi et al., 2023). Even so, the mechanism of myocardial fibrosis caused by COVID-19 is elusive. Elucidating the mechanisms of myocardial fibrosis caused by COVID-19 is imperative to prevent and treat COVID-19-associated myocardial fibrosis, thereby reducing the occurrence of malignant events. We summarize the latent mechanisms of myocardial fibrosis induced by COVID-19 and probe into promising prevention and treatment approaches. On this basis, we discuss possible future research directions in the post-COVID-19 era.

## 2 Underlying molecular mechanisms of COVID-19-associated myocardial fibrosis

**Figure 1:**
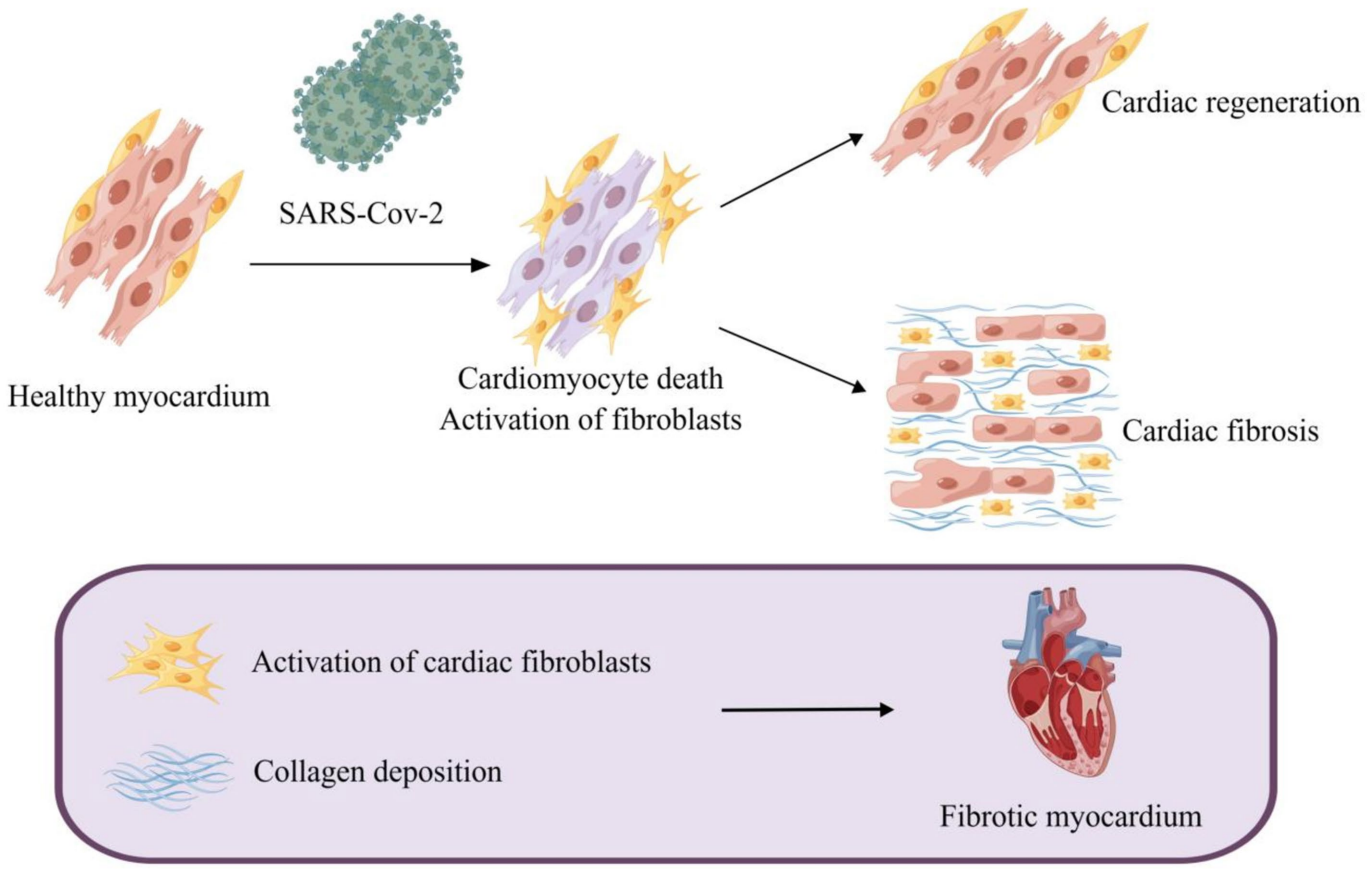
Myocardial fibrosis caused by SARS-CoV-2. SARS-CoV-2-induced cardiomyocyte death leads to activation of myofibroblasts and a reparative fibrotic response in the injured region. Activation of cardiac fibroblasts and deposition of collagen eventually lead to myocardial fibrosis.

The mechanism of myocardial fibrosis is extremely sophisticated. Myocardial cellular death is usually the initiating incident leading to the activation of the fibrogenic signaling pathway in the myocardium. In other circumstances, hazardous stimulating factors, myocardial inflammation, for example, may affect fibrosis without cell death (Kong et al., 2014). SARS-CoV-2, namely COVID-19, can conduce to the occurrence of myocardial fibrosis through both cardiomyocyte death-dependent and non-cardiomyocyte death-dependent pathways. Cardiac fibroblasts, one of the most abundant cell types in the heart, are considered primary target cells for the progression of myocardial fibrosis (Porter and Turner, 2009). As proof, cardiac fibrosis is characterized by the activation of cardiac fibroblasts (Travers et al., 2016) and collagen deposition (Figure 1; Ricard-Blum et al., 2018).

### 2.1 SARS-CoV-2 activates cardiac fibroblasts through multiple approaches

An army of factors affects the activation of cardiac fibroblasts, comprising angiotensin II (Ang II) (Crabos et al., 1994), aldosterone (Sakamuri et al., 2016), TGF-β1 (Liu et al., 2021), non-coding RNAs (Thum et al., 2008; van Rooij et al., 2008), glycolysis (Chen et al., 2021), modification of mitochondrial metabolism (Lombardi et al., 2019), connective tissue growth factor (CTGF) (Hong et al., 2018), and mechanical stress (van Putten et al., 2016).

#### 2.1.1 Renin-angiotensin-aldosterone system

**Figure 2:**
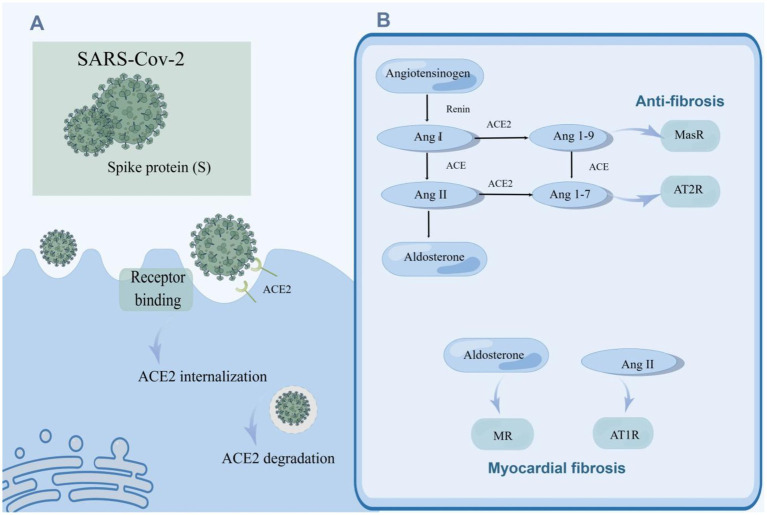
SARS-CoV-2 promotes myocardial fibrosis by downregulating ACE2 activity and causing an imbalance in the RAAS system. **(A)** The invasion of SRS-Cov-2 reduced ACE2 activity. SARS-CoV-2 enters the host cell through spike (S) binding with ACE2 on the surface of the host cell, and ACE2 is internalized. ACE2 can be degraded after entering cells. **(B)** SARS-CoV-2 caused an imbalance of the RAAS system which gives rise to myocardial fibrosis. Angiotensinogen is hydrolyzed to Ang I by renin, and ACE subsequently hydrolyzes Ang I to Ang II which enhances the secretion of aldosterone by the adrenal gland. Ang I and Ang II can be hydrolyzed to Ang 1–7 and Ang 1–9 by ACE2. Ang II and aldosterone facilitate myocardial fibrosis, while Ang 1–7 and Ang 1–9 alleviate myocardial fibrosis. SARS-CoV-2 can increase the levels of Ang II and aldosterone, and down-regulate the concentrations of Ang 1–7 and Ang 1–9 by suppressing the activity of ACE2, contributing to myocardial fibrosis.

**Figure 3:**
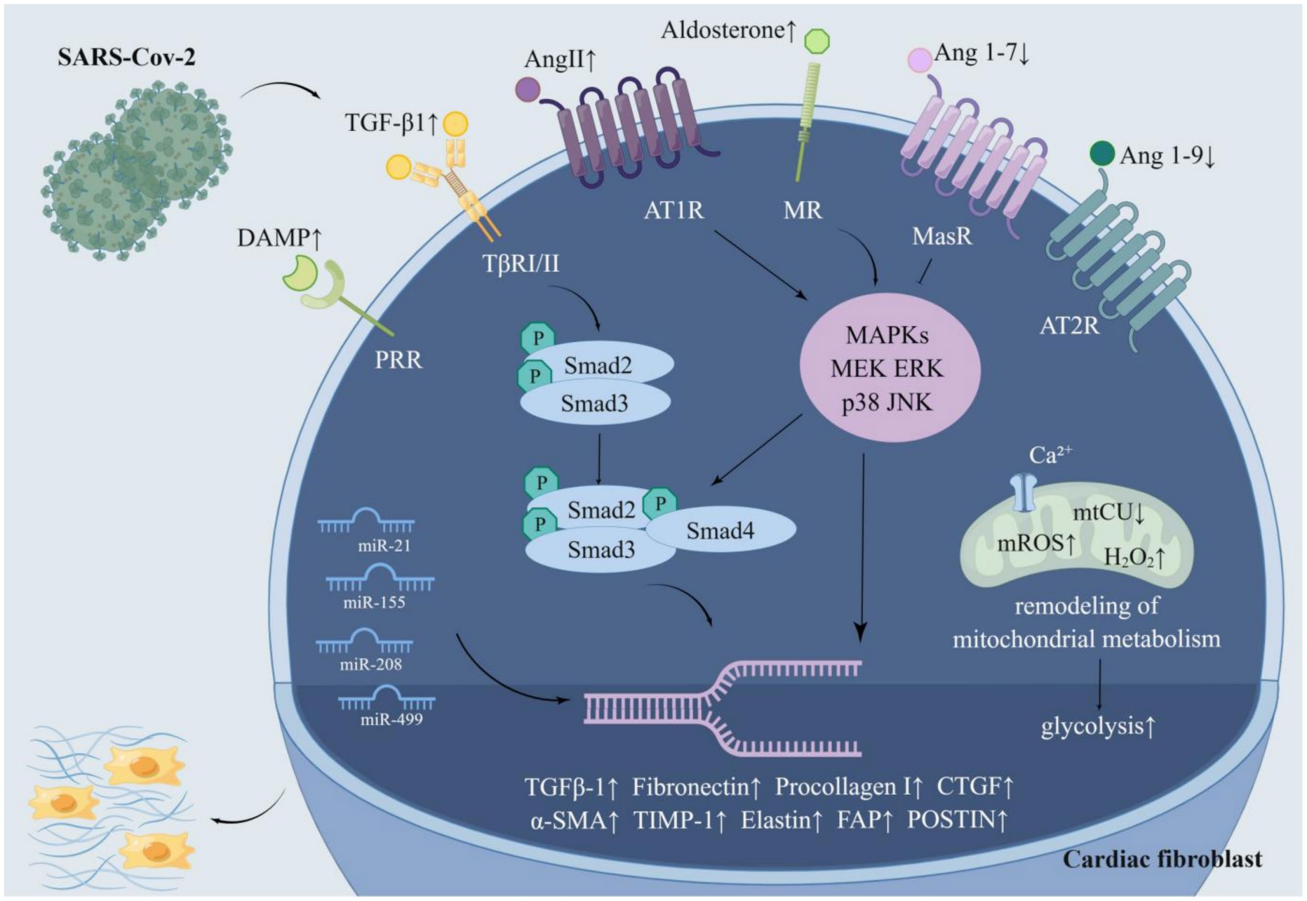
The molecular pathway of SARS-CoV-2 activating cardiac fibroblasts. SARS-CoV-2 activates cardiac fibroblasts in multifarious ways. SARS-CoV-2 can multiply the fibrogenic substances Ang II, aldosterone, DAMP, TGF-β 1, miR-21, miR-155, miR-20, and miR-499 while impairing the antifibrotic substances Ang 1–7, Ang 1–9 and miR-29. Furthermore, SARS-CoV-2 facilitates glycolysis and mitochondrial metabolic remodeling to accelerate myocardial fibrosis. Ang II and aldosterone activate the MAPK/ERK pathway and phosphorylate Smad2 and Smad3, which bind to Smad4 to form a complex. The complex is transported to the nucleus and then increased the expression of genes conducive to collagen deposition. Ang 1–7 and ang1-9, which are decreased in COVID-19, alleviate this process. SARS-CoV-2 resulted in a remarked enhancement of TGF-β1, which boosts the expression of related genes through the classical TGF-β1/Smad pathway. SARS-CoV-2 may reduce mitochondrial calcium uptake by destroying mtCU, which in turn brings about mitochondrial metabolic remodeling and promotes myocardial fibrosis.

RAAS is an essential regulatory system consisting of a series of peptide hormones and corresponding enzymes. They widely exist in a variety of organ tissues, including myocardium and vascular smooth muscle, and participate in the regulation of target organs together (Patel et al., 2017). Renin can hydrolyze angiotensinogen to produce the decapeptide angiotensin I (Ang I) (Hamada and Kiso, 2016). Ang II is generated by the hydrolysis of Ang I with the action of angiotensin-converting enzyme (ACE) and is the main effector substance of RAAS (Mehta and Griendling, 2007; Marchesi et al., 2008). Ang II works by binding to highly specific receptors on the surface of the cell membrane. Ang II receptors are divided into two major subtypes: angiotensin II type 1 receptor (AT1R) and angiotensin II type 2 receptor (AT2R) (Timmermans et al., 1992; Akazawa et al., 2013). Ang II is an important molecule that promotes myocardial fibrosis, and its pro-fibrosis effect is produced by activating AT1R (Kim et al., 1995; Figure 2B). AT1R is a G protein-coupled receptor. Ang II and AT1R binding activates the mitogen-activated protein kinase (MAPK)/ERK pathway and phosphorylates Smad2 and Smad3 (Xu et al., 2018). Phosphorylated Smad2 and Smad3 bind to Smad4 to form a complex that will be transported to the nucleus, causing transcription of TGF-*β*, fibronectin, and procollagen I genes (Tian et al., 2020; Martin et al., 2015; Wong et al., 2018; Figure 3). Additionally, upon binding of Ang II to AT1R, phosphorylation of Jak2 and Tyk2 occurs rapidly, leading to phosphorylation of members of the signal transducer and activator of the transcription (STAT) family of transcription factors. Ang II has been reported to facilitate the nuclear translocation of STAT1 in cardiac fibroblasts of neonatal rats (Marrero et al., 1995). Coincidentally, in a retrospective study involving 82 COVID-19 patients and 12 individuals not infected by SARS-CoV-2, the plasma Ang II level of critical patients sly higher than that of mild patients and control groups. Univariate analysis manifested an association between Ang II levels and disease severity. However, this study showed no significant difference in plasma renin levels between the COVID-19 sufferers and the control group, indicating that the enhancement of Ang II may not have been caused by an increase in renin (Wu et al., 2020). Interestingly, in another study renin inhibitors had the same effect as ACE inhibitors (ACEIs) and angiotensin II type 1 receptor blockers (ARBs) in the treatment of COVID-19 (Lumbers et al., 2022). This paradox may be related to changes in renin concentration throughout the disease, and the underlying mechanisms can be further clarified by monitoring renin concentrations at different periods of the disease. Surprisingly, the researchers discovered that increased Ang II concentrations can be caused by decreased metabolism. ACE2 mRNA and cell surface ACE2 expression decreased during COVID-19, Ang II was metabolized to a lesser extent by ACE2, and its plasma concentration increased (Lombardi et al., 2019). In conclusion, the imbalance of RAS in COVID-19 leads to cellular fibrosis through virus-mediated downregulation of ACE2 in favor of the pro-fibrotic ACE/Ang II/AT1R axis (Khazaal et al., 2022).

ACE2, a member of the RAAS, is closely associated with SARS-CoV-2 infection. ACE2 plays a key role not only in viral entry but also in preventing acute cardiovascular injury (Li et al., 2020b; Hoffmann et al., 2020; Gheblawi et al., 2020). The entry of SARS-CoV-2 into host cells is mediated by the binding of the glycoprotein spike (S) protruding from the viruses surface (Walls et al., 2020) to ACE2 located on the cell surface, which is widely distributed and abundantly expressed in the lungs, blood vessels, and heart (Hoffmann et al., 2020). It is then internalized and broken down, leading to a decrease in its activity on the cell surface (Datta et al., 2020; Figure 2A). Its extracellular portion is released into the extracellular fluid to form soluble ACE2 (sACE2) which is used to bind circulating viral particles, thereby reducing the infectivity of SARS-CoV-2 (Zipeto et al., 2020). Contrary to this, another study demonstrated a positive correlation between sACE2 activity and the severity of COVID-19 (Fagyas et al., 2022). This discrepancy may be due to recording data at different stages of the disease course. Meanwhile, ACE2 mRNA expression was also inhibited after infection with SARS-CoV-2 (Oudit et al., 2009). On the other hand, ACE2 is responsible for the hydrolyzation of Ang I and Ang II into angiotensin 1–9 (Ang 1–9) and angiotensin 1–7 (Ang 1–7), respectively (Liao and Wu, 2021). The downregulation of ACE2 activity accounts for the elevation of the circulating Ang II level and the decrease of Ang 1–7 and Ang 1–9. Ang 1–7 binds to a specific receptor, MasR, which is also a G protein-coupled seven-transmembrane protein. Ang 1–7 antagonizes metabolic disorders associated with cardiac fibroblast activation arising from Ang II (Figure 2B). Studies have shown that Ang 1–7 can attenuate the chronic stimulation of MAPK, ERK1/2, p38, and JNK which promote fibrosis (Alzayadneh and Chappell, 2014; Meng et al., 2014). Furthermore, Ang 1–7 suppresses Smad phosphorylation and inhibits the expression of collagen, CTGF, and *α*-SMA (α-smooth muscle actin) (Cai et al., 2016). Ang 1–9, an endogenous biological inhibitor of Ang II binds to AT2R, a G protein-coupled receptor. In contrast to AT1R, ERK1/2 activity was weakened when AT2R was activated (Faria-Costa et al., 2014). Studies have displayed that Ang 1–9 could alleviate cardiac fibrosis by inhibiting the proliferation of fibroblasts and regulating the expression of collagen I (Flores-Munoz et al., 2012; Figure 3). In a word, ACE2 activity will be impaired after SARS-CoV-2 infection, contributing to a decrease in the antifibrotic substances produced by ACE2 hydrolysis and an increase in the substrate, i.e., fibroblastic substances (Figure 2B). It is worth mentioning that ACE2 may play a dual role in COVID-19-associated myocardial fibrosis because it mediates SARS-CoV-2 into cardiac cells and causes cardiac injury (Vitiello and Ferrara, 2020).

Ang II promotes aldosterone secretion from the adrenal cortex. Thus, the level of aldosterone will consequentially increase with the increment of Ang II caused by COVID-19. Aldosterone acts by binding to the mineralocorticoid receptor (MR) (Figure 2B; Lother et al., 2015). MR is expressed in many tissues/cell types such as kidney, heart, and fibroblasts, and pathological MR hyperactivation leads to inflammation and fibrosis, which can directly induce damage to target organs such as the heart and kidney in animal models (Agarwal et al., 2021; Cohen et al., 2023; Bauersachs et al., 2015). Studies demonstrated that aldosterone could stimulate collagen synthesis in a dose-dependent manner. Spironolactone, an aldosterone receptor antagonist, eliminated the aldosterone-mediated increase in collagen synthesis, further indicating the fibrogenic effect of aldosterone (Brilla, 2000). Moreover, aldosterone induces the expression of TIMP-1 (tissue inhibitor of matrix metalloproteinases-1) and further promotes the accumulation of collagen (Hung et al., 2016). Aldosterone-induced fibroblast proliferation also requires signal transduction through PI3K, JNK, and ERK pathways (Huang et al., 2012). Above all, RAAS plays an essential role in COVID-19-related myocardial fibrosis (Figure 2B), which lays the foundation for the application of RAAS inhibitors to the treatment of COVID-19-related myocardial fibrosis.

#### 2.1.2 Transforming growth factor-*β*1

TGF-β1 is undoubtedly one of the most relevant pro-fibrotic factors (Vallee and Lecarpentier, 2019; Meng et al., 2016). TGF-β1 binds to serine–threonine kinase receptor type I (TβRI) (Franzén et al., 1993) and receptor type II (TβRII)(Lin et al., 1992) to form a heterotetramer that specifically induces phosphorylation of Smad2 and Smad3 (Budi et al., 2017; Kamato et al., 2013). Phosphorylated Smad2 and Smad3 form transcriptional complexes with Smad4 and translocate to the nucleus thereby mediating the expression of target genes to activate cardiac fibroblasts (Ten Dijke and Hill, 2004; Kawabata et al., 1999). TGF-β1 also activates cardiac fibroblasts through non-Smad signaling pathways (Zhang, 2017; Mu et al., 2012). Ghazavi et al. (2021) conducted a study among 63 adult COVID-19 patients and compared them with 33 age-and sex-matched healthy subjects as controls. They revealed that serum TGF-β levels were significantly elevated in COVID-19 patients compared to healthy subjects. Another research found that serum TGF-β levels were significantly higher in patients with severe COVID-19 compared to those with mild disease and identified TGF-β as a potential predictor of prognosis in patients with COVID-19 (Mustroph et al., 2021). Elevated TGF-β1 levels and Smad3 profiling were also found in cardiac cadaveric samples from COVID-19 patients (Mustroph et al., 2021). Furthermore, in a minimally invasive autopsy trial of six patients who died from COVID-19, elevated TGF-*β* expression and elevated collagen in the interstitial and perivascular spaces of the samples were found, which suggested myocardial fibrosis (Hartmann et al., 2021). Accordingly, it can be hypothesized that SARA-Cov-2 could facilitate cardiac fibrosis by raising TGF-β1 levels in infected patients to stimulate cardiac fibroblasts (Figure 3). In particular, the concept of PPCS (persistent post-COVID syndrome) was introduced recently to describe the persistent sequelae after COVID-19, including persistent lung, heart, and blood vessel fibrosis and immunosuppression (Oronsky et al., 2021). In addition to its pro-fibrotic function, TGF-β1 has an immunosuppressive effect (Sheng et al., 2015). This is why TGF-β1 is elevated during the prolonged immunosuppressive phase after the onset of COVID-19 (Colarusso et al., 2021). This suggests that TGF-β1 may still promote systemic organ fibrosis via enhanced TGF-β1 after recovery from COVID-19. Therefore, inhibition of TGF-β1 expression may alleviate immunosuppression and fibrosis in the lung, heart, and blood vessels after COVID-19.

#### 2.1.3 microRNAs

microRNAs (miRNAs/miRs), small non-coding RNA molecules (Mohr and Mott, 2015), were reported to impact the activation of cardiac fibroblasts (Li C. et al., 2021). To illustrate, miR-21 activates the ERK–MAPK signaling pathway in cardiac fibroblasts by inhibiting sprouty homolog 1. This mechanism makes a difference in fibroblast survival and growth factor secretion and governs the extent of myocardial fibrosis (Thum et al., 2008). A study found that the circulating and local miR-21 levels in patients with myocardial fibrosis were remarkably multiplied, and emphasized the value of circulating miR-21 as a biomarker of myocardial fibrosis (Villar et al., 2013). Concentrations of miR-21 were measured by miR-specific TaqMan PCR analysis, revealing that the levels of miR-21 in the sera of COVID-19 patients were significantly elevated (Garg et al., 2021; Tang et al., 2020). miR-155 activates cardiac fibrosis through the nuclear factor erythroid-2-related factor 2/HO-1 (heme oxygenase-1) signaling pathway (Li et al., 2020a) and the cellular c-Ski (Sloan-Kettering Institute)/Smad pathway and boosts the production of ECM (Wang F. et al., 2021). Similarly, miR-208 and miR-499 activate cardiac fibroblasts and enhance myocardial fibrosis as well (Corsten et al., 2010; Sun et al., 2021). It’s extensively validated that the serum levels of miR-155, miR-208, and miR-499 are much greater in COVID-19 patients compared to healthy individuals (Garg et al., 2021; Donyavi et al., 2021; Kassif-Lerner et al., 2022). Therefore, the above miRNAs may be a nexus with the activation of cardiac fibroblasts. Besides, miR-29 expression in cardiac tissues was 5 to 12 times more abundant than in other tissues. Its reduction activates the TGF-β pathway, which favors fibroblast proliferation and induces the expression of collagen I and collagen III, elastin, and fibronectin. These changes increase the deposition of components in the ECM, leading to myocardial stiffness and diastolic dysfunction (Zhu et al., 2013; Abonnenc et al., 2013; Boon et al., 2011). Coincidentally, miR-29 serum levels were notably diminished in COVID-19 sufferers (Keikha et al., 2021). This suggests that SARS-CoV-2 may contribute to the development of myocardial fibrosis by decreasing miR-29 levels (Figure 3).

#### 2.1.4 Glycolysis

It was demonstrated that cardiac fibrosis is frequently accompanied by enhanced glycolysis and that inhibition of glycolysis alleviates cardiac fibrosis after infarction (Chen et al., 2021). *In vitro* experiments proved that SARS-CoV-2 resulted in considerable alterations in protein omics, with the majority of the significantly elevated proteins belonging to glycolysis-related proteins. The metabolites such as pyruvic acid and lactic acid were also increased (Krishnan et al., 2021). It was demonstrated by experiments in the Drosophila heart that SARS-CoV-2 binds to the host protein MGA/MAX complex, resulting in the activation of the glycolytic process. This process was also found in mouse cardiomyocytes (Zhu et al., 2022). SARS-CoV-2 could foster glycolysis by influencing the expression of glucose transporters and the AKT/mTOR/HIF-1 signaling pathway (Appelberg et al., 2020). Hence, SARS-CoV-2 may hasten myocardial fibrosis by speeding up glycolysis (Figure 3).

#### 2.1.5 Mitochondrial metabolism

Recent studies revealed that mitochondria are key regulators of myocardial fibrosis, which promotes metabolic remodeling by reducing mitochondrial Ca^2+^ (mCa^2+^) uptake. mCa^2+^ signaling is a regulatory mechanism that integrates fibroblast differentiation and fibrosis (Lombardi et al., 2019). The mitochondrial calcium uniporter (MCU) alters gating in a MICU1 (mitochondrial calcium uptake 1)-dependent manner, which could reduce mCa^2+^ uptake and induce coordinated changes in metabolism, including heightened glycolysis. Mitochondria also augment mitochondrial reactive oxygen species (ROS) production, which drives epigenetic changes in fibroblasts, leading to epigenetic reprogramming of fibroblasts to myofibroblasts (Jain et al., 2013; Guido et al., 2012; Lu et al., 2014). Furthermore, mitochondria suppress apoptotic pathways in response to pro-fibrotic stimuli and the state of terminal differentiation, sustaining the persistence of myofibroblasts, which contributes to the progression of cardiovascular disease (Cai et al., 2019; Gibb et al., 2020; Dong et al., 2014). It was shown that spiny proteins on the SARS-CoV-2 surface induced transcriptional suppression of mitochondrial metabolic genes, leading to myocardial fibrosis in mice (Cao X. et al., 2023). Down-regulation of mitochondrial genes further induced the expression of microRNA 2392 and activated HIF-1α to stimulate glycolysis (Guarnieri et al., 2023). More importantly, there was a mtCU degradation mediated by immunity and the rise of H_2_O_2_ in mitochondria in AT-II cells (alveolar type II epithelial cells, AT-II) of mice with ARDS (Islam et al., 2021). In conclusion, SARS-CoV-2 may conduce to metabolic remodeling of cardiac fibroblasts through alterations in mitochondrial metabolism (Figure 3).

#### 2.1.6 Damage-associated molecular pattern

Cardiac fibroblasts express a series of innate immune pattern recognition receptors. These receptors activate cardiac fibroblasts upon binding to an assortment of DAMPs (Humeres and Frangogiannis, 2019). Recent plasma proteomics studies indicated that DAMP is elevated in patients with moderate to severe COVID-19, including circulating mitochondrial DNA, HMGB1, and S100B proteins (Parthasarathy et al., 2022; Silvin et al., 2020; Aceti et al., 2020; Scozzi et al., 2021). It can be hypothesized that SARS-CoV-2 activates cardiac fibroblasts by increasing circulating and local cardiac DAMP concentrations (Figure 3).

It is worth noting that the above mechanisms are not self-contained, but rather constitute a complicated network. For instance, the fibrogenic effect of Ang II is largely mediated by TGF-*β* signaling (Garvin et al., 2021). Real-time PCR in Ang-II-treated hearts revealed a significant increase in TGF-β1 (Haudek et al., 2010). And MFGE8 (milk fat globule-EGF factor 8) attenuates Ang II-induced myocardial fibrosis by inhibiting the TGF-β1/Smad2/Smad3 pathway (Ge et al., 2020). Besides, Ang II induces ROS production and thus stimulates fibroblast activation (Wang et al., 2001). Ang II significantly increased the activity of NAD (P) H oxidase, which is the key enzyme to produce ROS (Griendling et al., 1994). The inhibitory effect of NAD(P)H oxidase complex on collagen production stimulated by Ang II in cardiac fibroblasts of adult rats *in vitro* can be observed (Lijnen et al., 2006). Meanwhile, the accumulation of ROS leads to NLRP3 inflammatory vesicle activation and IL-1β production, which initiates a signaling cascade response that upregulates TGF-β1 transcription and causes myocardial fibrosis (Van Tin et al., 2024). What’s more, research illuminated that interleukin (IL)-6 signal transduction contributed to aldosterone-induced cardiac fibrosis in cardiac fibroblasts (Chou et al., 2018). And IL-6 might be the crucial mediator of macrophage recruitment and infiltration in cardiac fibro originating ates from aldosterone (Liao et al., 2020). Inflammation also participated in Ang II-dependent myocardial fibrosis (Qi et al., 2019). Accumulating studies demonstrated the interaction of miR-29 and the TGF-β/Smad pathway (Van Rooij et al., 2008; Qin et al., 2018). TGF-β weakened the expression of miR-29 by binding to a site on the miR-29 promoter, thereby showing a pro-fibrosis effect (Zhu et al., 2013; Divakaran et al., 2009). In short, SARS-CoV-2 activates cardiac fibroblasts and promotes myocardial fibrosis through the above complex network.

### 2.2 Manifestations of fibroblast activation in COVID-19

Cardiac fibroblasts undergo a range of changes upon activation, such as proliferation, augmented expression of periostin (POSTN), and differentiation into myofibroblasts (Snider et al., 2009). Cardiac autopsy of COVID-19 patients detected an enlarged proportion of cardiac fibroblasts, suggesting that cardiac fibroblasts proliferate intensively in COVID-19 victims. POSTN, a matricellular protein, the distribution, and expression of which is coherent with the degree of myocardial fibrosis, plays a pivotal role in cardiac development and remodeling. Fibroblast activation protein is difficult to detect in disease-free adult organs, while it is substantially elevated in tissues undergoing remodeling. Single nucleus RNA sequencing displayed that the expression of FAP (fibroblast activation protein) and POSTN, markers of activated fibroblasts, was increased in cardiac fibroblasts from COVID-19 sufferers (Delorey et al., 2021). Single-cell transcriptome analysis revealed a shift from fibroblasts to myofibroblasts in the lungs of COVID-19 patients (Bharat et al., 2020). Similar changes may have occurred in the heart, although there are no relevant studies. In summary, cardiac fibroblasts from COVID-19 patients underwent a series of post-activation alterations (Figure 3).

## 3 Role of other cells in COVID-19-associated myocardial fibrosis

Besides cardiac fibroblasts, a broad range of other cells is also engaged in the process of myocardial fibrosis. Cardiomyocytes, endothelial cells, and diverse immune cells in the heart can secrete pro-fibrotic factors to activate cardiac fibroblasts (Lopez et al., 2021). Bioinformatics analysis of single-cell transcriptional data from cardiac non-myocytes has confirmed that cardiac fibroblasts establish a rich intercellular communication network with other cell populations like endothelial cells, glial cells, and macrophages (Skelly et al., 2018; Farbehi et al., 2019). SARS-CoV-2 can contribute to the incidence of myocardial fibrosis through a variety of cells.

### 3.1 Immunocytes and inflammation

**Figure 4:**
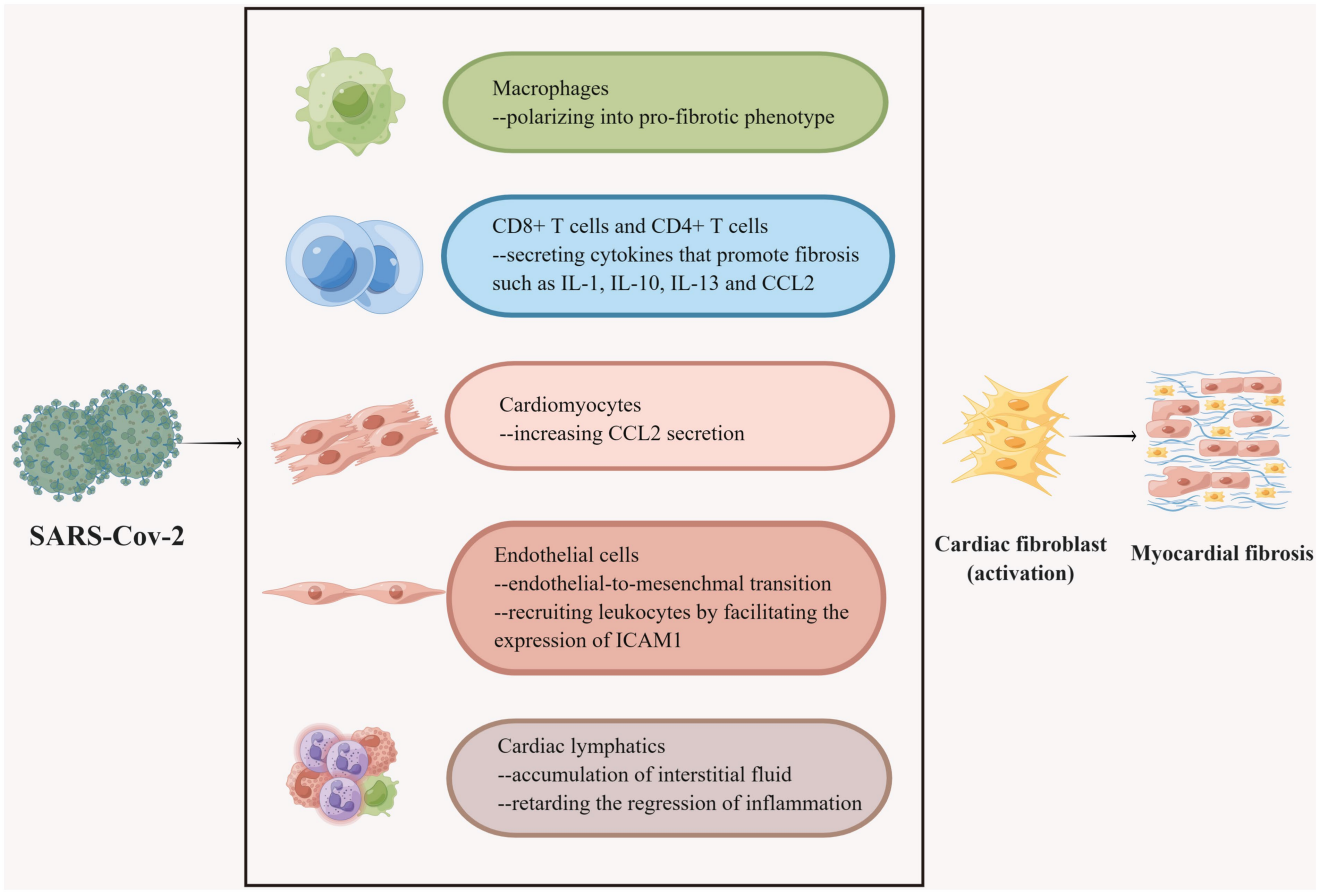
Multiple cells participate in COVID-19-associated myocardial fibrosis. Macrophages have different phenotypes and SARS-CoV-2 promotes the polarization of macrophages into fibrogenic phenotypes. CD8 + T cells and CD4 + T cells secrete a variety of fibrogenic cytokines under the stimulation of the virus. Myocardial cells secrete CCL2, recruit leukocytes, and promote fibrosis after being invaded by SARS-CoV-2. SARS-CoV-2 invasion can induce endothelial cells to undergo EndMT, which is one of the sources of cardiac fibroblasts. SARS-CoV-2 can also boost endothelial cells to express ICAM-1 to recruit leukocytes and further accelerate fibrosis. SARS-CoV-2 is severely detrimental to cardiac lymphatic vessels, which accounts for the accumulation of cardiac interstitial fluid and slows down the regression of inflammation, thus resulting in myocardial fibrosis.

Inflammation and fibrosis are intertwined networks. As a subtype of tissue-resident immune cells, CXCL8^hi^CCR2^+^HLA-DR^hi^ macrophages preferentially localize to severely fibrotic myocardium and drive leukocyte recruitment and inflammation (Rao et al., 2021). In an experiment with SARS-CoV-2-infected nonhuman primates (NHPs), myocardial tissue samples were found by specific immunohistochemical staining to show multiple T lymphocytes and macrophage foci and myocardial fibrosis in the cardiac interstitium and perivascular regions of infected animals (Rabbani et al., 2022). As NHPs are physiologically similar to humans, it can be hypothesized that a similar process occurs in COVID-19 patients (Petkov et al., 2019). Single-cell analysis of cardiac tissue specimens from COVID-19 patients demonstrated an enlarged proportion of macrophages (Delorey et al., 2021). Of note, macrophages can be polarized into a pro-fibrotic phenotype under certain circumstances. SARS-CoV-2 was proven to facilitate the transition of macrophages to a pro-fibrotic transcriptional phenotype in the lungs of COVID-19 individuals (Wendisch et al., 2021). Analogous transformations may occur in the heart (Figure 4). Likewise, it was widely validated that CD8+ T cells and CD4+ T cells infiltrate the heart when fibrosis develops (Rao et al., 2021; Bansal et al., 2017). T cells have a hand in the progression of cardiac fibrosis (Nevers et al., 2017). Coincidentally, multifocal lymphocytic myocarditis was noted in cardiac samples from patients with COVID-19 (Basso et al., 2020; Buja et al., 2020). What’s more, a multitude of cytokines, growth factors, and chemokines secreted by leukocytes can expedite fibrogenesis (Figure 4). For instance, the chemokine ligand (CCL2) stimulates fibroblasts to proliferate and differentiate into myofibroblasts (Kruglov et al., 2006). In addition, the chemokine CCL7 in turn promotes leukocyte aggregation to sites of infection/inflammation (Tsou et al., 2007). In a recent study, Ćorović et al. found that COVID-19-associated myocardial fibrosis was associated with immunodysregulation of CD8+ T cells, CD8+ T effector memory (TEM), and CD4+ Th2-like cells by integrating clinical imaging and immunophenotyping (Corovic et al., 2024). Moreover, increased circulating CCL7 may promote cytotoxic CD8+ T cell recruitment to the heart, with potentially deleterious effects on cardiac remodeling after COVID-19. It has been revealed that elevated blood CCL7 levels and decreased CD8+ TEM cell counts were the most clinically characterized markers predicting COVID-19-related cardiac MRI abnormalities (Corovic et al., 2024). Nevertheless, due to the relatively small sample size and the disproportionality of the included study subjects, smaller subgroup analyses could not be performed, and more in-depth studies on the mechanism of COVID-19-associated myocardial injury are needed.

IL-1, IL-4, IL-10, and IL-13 modulate the phenotypic conversion of cardiac fibroblasts to synthesize ECM or secrete matrix metalloproteinases (Frangogiannis, 2019). In addition, neutralizing IL-6 was demonstrated to reduce cardiac fibroblast activation in mice. MAPK and CaMKII (Ca^2+^/calmodulin-dependent protein kinase II)-STAT3 was inhibited in cardiac fibroblasts following the neutralization of IL-6 (Kumar et al., 2019). It is reported that a 44-year-old woman with COVID-19 was admitted to the hospital with complaints of chest pain; cardiac magnetic resonance and myocardial biopsy were suggestive of myocardial fibrosis, and normal CRP levels suggested that IL-6 was involved in this process (Usui et al., 2023). IL-1*β* plays a role in promoting fibrosis by binding to IL-1βR (Mack, 2018). COVID-19 could cause a cytokine storm in some patients, defined as an excessive immune response to external stimuli, characterized by pathologically elevated cytokine levels. COVID-19 individuals have high amounts of the aforementioned cytokines and chemokines in their blood, which induce fibrosis in many organs throughout the body and perpetuate the disease (Kounis et al., 2024; Kim et al., 2021). IL-33 is also a member of the IL-1 family and is secreted by cardiac fibroblasts to respond to myocardial strain or injury (Cayrol and Girard, 2018; Zhang et al., 2021). The interaction between IL-33 and the transmembrane form of ST2 (ST2L) is a part of the cardiac protective pathway, preventing myocardial fibrosis and weakening inflammatory reaction, hypertrophy, and apoptosis (Vianello et al., 2019). ST2L/IL-33 signaling is adjusted by the soluble ST2 (sST2), which is mainly secreted by resident cardiac fibroblasts. When circulating levels of sST2 are aberrantly augmented, the tight binding of sST2 to IL-33 results in the deactivation of ST2L/IL-33 and contributes to pro-fibrotic and pro-apoptotic signaling and ultimately leads to interstitial cardiac fibrosis and myocyte hypertrophy. Therefore, sST2 serves as a biomarker of cardiac stress and fibrosis (Vianello et al., 2019; Miftode et al., 2021). According to considerable research, a high level of serum sST2 was detected in COVID-19 patients without cardiovascular comorbidities, and it has been highlighted as a valuable prognostic factor for these patients (Zeng et al., 2020). This indicates that sST2 may, to some extent, impinge on the prognosis of COVID-19 patients by prompting myocardial fibrosis. In addition, activation of transient receptor potential anchor protein 1 (TRPA1) expressed in the cardiovascular system induces inflammation and oxidative stress, which in turn leads to the formation of COVID-19 sequelae (Zhao et al., 2023), and blockade of TRPA1 reduces the expression of pro-fibrotic cytokines and ameliorates cardiac fibrosis (Wang et al., 2018). Therefore, it can be hypothesized that TRPA1 plays an important role in forming COVID-19-associated myocardial fibrosis, which provides a potential drug target for treating COVID-19-associated myocardial fibrosis.

### 3.2 Endothelial cells

The viral entry receptor ACE2 on the surface of endothelial cells is highly expressed and susceptible to infection by SARS-CoV-2 (Varga et al., 2020). Endothelial-to-mesenchymal transition (EndMT) is a transformation process in which the morphology, phenotype, and function of endothelial cells are distinctly altered physiologically or pathologically, assuming the characteristics of fibroblasts. EndMT participates in the process of myocardial fibrosis (Zeisberg et al., 2007; Gong et al., 2017). SARS-CoV-2 stimulates the expression of TGFs, IL-1 and IL-6, which are known to trigger EndMT in endothelial cells (Huang Y. et al., 2020; Huang C. et al., 2020; Maleszewska et al., 2013; Takagaki et al., 2020). In particular, the main inducer of EndMT, TGF-β, can be mediated by miR-21 and NF-κB in this process (Kumarswamy et al., 2012; Maleszewska et al., 2013). As mentioned earlier, the level of miR-21 in patients with COVID-19 is elevated. It has been shown that the level of NF-κB was elevated in the cardiac tissues of SARS-CoV-2 Spike protein-treated mice (Liang S. et al., 2023). Therefore, it can be hypothesized that SARS-CoV-2 promotes myocardial fibrosis by driving EndMT. Besides, endothelial cells recruit leukocytes by facilitating the expression of intercellular adhesion molecule 1 (ICAM-1), which secretes pro-fibrotic factors that activate fibroblasts, thereby promoting fibrosis in the heart (Shi et al., 2010; Kanters et al., 2008). The ICAM1 gene was found to be upregulated in endothelial cells in lung endothelial cells of COVID-19 victims and was associated with immune hyperactivation in the lungs and circulation (Birnhuber et al., 2021; Qin et al., 2021). The same process occurs in the hearts of COVID-19 individuals. Increased expression of ICAM-1 was found in post-mortem biopsies of the myocardium of COVID-19 patients, as well as increased indicators of myocardial fibrosis compared to controls (Hartmann et al., 2021). Notably, cardiac autopsy in COVID-19 subjects uncovered an incremental percentage of endothelial cells in cardiac tissue compared to normal subjects, which may also be attributable to myocardial fibrosis (Figure 4) (Delorey et al., 2021).

### 3.3 Pericyte

Pericytes with ACE-2 receptors can also serve as targets of SARS-CoV-2 (Sakamoto et al., 2021). Damage to virus-infected pericytes leads to impaired microcirculation, further contributing to inflammation and cardiac fibrosis, as well as susceptibility to NF-κB-induced cell death (Chen L. et al., 2020; Frangogiannis, 2024; Khan et al., 2022). Several studies demonstrated that SARS-CoV-2 infection degraded the glycocalyx, a major component of the endothelial filtration barrier, as well as pericyte disintegration, which severely compromised the integrity of lymphatic endothelial cells and the endothelial barrier, and amplified its permeability (Varga et al., 2020; Amri et al., 2022; Rauch et al., 2020; Chioh et al., 2021; Panagiotides et al., 2024). Malfunction of the lymphatic endothelium retards the regression of inflammation (Brakenhielm and Alitalo, 2019), which has a significant effect on the development of diffuse myocardial fibrosis as previously described (Figure 4). Moreover, impairment or dysfunction of the cardiac lymphatics can lead to the o accumulation of interstitial fluid (i.e., edema) and the advancement of diffuse myocardial fibrosis (Panagiotides et al., 2024; Brakenhielm et al., 2020).

### 3.4 Cardiomyocytes

It was shown that SARS-CoV-2 infected cardiomyocytes *in vitro* in an ACE 2 and cathepsin-dependent manner (Bojkova et al., 2020b; Bailey et al., 2021). Moreover, human cardiomyocytes exposed to SARS-CoV-2 increased CCL2 secretion, recruiting monocytes to the site of infection (Yang et al., 2021), and the monocyte recruitment-associated protein CCL-2/MCP-1 was linked to fibrosis in a cohort study (Holton et al., 2023). Meanwhile, CCL2 led to monocyte recruitment (Tsou et al., 2007). In another study expanded CCL2 expression and macrophage infiltration were indeed observed in the hearts of SARS-CoV-2-infected hamsters (Chen S. et al., 2020). This crosstalk further boosts myocardial fibrosis (Figure 4).

## 4 Promising prevention and reversal therapy for COVID-19-related myocardial fibrosis

The clinical management strategy for COVID-19 patients is sophisticated. Currently, deployed drugs are primarily anti-retroviral drugs and immunomodulators targeting two phases of the SARS-CoV-2 pathogenesis, early viral replication, and late excessive immune/inflammatory response, respectively. In consideration of the above, it is of vital importance to adopt therapeutic strategies aimed at preventing and treating myocardial fibrosis due to COVID-19. Finding added value to current therapeutic solutions or adding antifibrotic agents is crucial.

Table 1 lists the clinical evidence for the efficacy of the drugs described below on COVID-19 and myocardial fibrosis.

**Table 1:** Latent drugs for the treatment of COVID-19-associated myocardial fibrosis.

Drugs	Mechanisms of Treating COVID-19 and myocardial fibrosis	Clinical trials or meta-analyses for treating COVID-19	Clinical evidence for treating myocardial fibrosis
Remdesivir	RNA polymerase inhibitor	Beigel et al. (2020) and Rosenberg (2021)	Li X. et al. (2021) and Xu et al. (2021)
Lisinopril	ACEI	No clinical trial	Chang et al. (2016) and Brilla et al. (2000)
Captopril	ACEI	No clinical trial	Rahman et al. (2010)
Telmisartan	ARB	Duarte et al. (2021)	Chang et al. (2016)
Candesartan	ARB	Lukito et al. (2021)	Ciulla et al. (2009)
Spironolactone	Aldosterone receptor blocker	Mareev et al. (2020)	Izawa et al. (2005) and Abbasi et al. (2022)
Tocilizumab	Antibody against the IL-6 receptor	Sheppard et al. (2017) and Lan et al. (2020)	Ishizaki et al. (2021)
Anakinra	IL-1 receptor antagonist	Bozzi et al. (2021)	Mezzaroma et al. (2015)
Colchicine	Anti-inflammatory and antiviral substance	Lopes et al. (2021) and Deftereos et al. (2020)	Chen Y. et al. (2020)

ACEI, angiotensin converting enzyme inhibitor; ARB, angiotensin II type 1 receptor blocker; COVID-19, indicates coronavirus disease 2019.

### 4.1 Antiviral drugs

Antiviral drugs can be divided into two types in mechanism: preventing viruses from entering host cells and inhibiting virus replication in cells. At present, most of the anti-SARS-CoV-2 drugs used are the latter (Majumder and Minko, 2021).

#### 4.1.1 Remdesivir

Remdesivir is an RNA polymerase inhibitor, which has effective antiviral activity *in vitro* and is effective in the COVID-19 animal model (Amirian and Levy, 2020; Williamson et al., 2020). For adults hospitalized with COVID-19 and with evidence of lower respiratory tract infection, remdesivir is superior to placebo in shortening the recovery time (Beigel et al., 2020; Rosenberg, 2021). The 28-day recovery rate, low-flow oxygen support from day 1 to day 14, and invasive mechanical ventilation or extracorporeal membrane oxygenation requirements from day 14 to day 28 of follow-up time in the remdesivir group were significantly improved. In addition, the risk of serious adverse reactions in the remdesivir group was markedly lower than in the control group (Rezagholizadeh et al., 2021). The therapeutic effect of fibrosis has been proved in the kidneys, lungs, and skin of animal models (Zhang et al., 2024; Li X. et al., 2021; Xu et al., 2021). An *in vitro* experiment used a mouse model of pulmonary fibrosis induced by bleomycin to evaluate the effect of fibrosis. The results showed that remdesivir inhibited the activation of pulmonary fibroblasts induced by TGF-*β*1 in a dose-dependent manner, improved the transformation of the alveolar epithelium into interstitial tissue, and alleviated collagen deposition (Li X. et al., 2021). In addition, the enhanced Smad3 phosphorylation was weakened by remdesivir in cellular and animal models of renal fibrosis. After remdesivir treatment *in vitro* and *in vivo*, the expression of anti-fibrosis factor Smad7 increased (Xu et al., 2021). At present, there is no research on the relationship between remdesivir and myocardial fibrosis, which can also be a future research direction.

### 4.2 Renin-angiotensin-aldosterone system inhibitors

As previously mentioned, RAAS performs a pivotal function in the pathogenesis of COVID-19-associated myocardial fibrosis. RAAS inhibitors, including ACEI and ARB, were widely employed in the treatment of hypertension, heart failure, and coronary artery disease (Ames et al., 2019).

#### 4.2.1 Angiotensin-converting enzyme inhibitor

ACEIs can reduce Ang II production by competitive conjugation to prevent the multiple pathways of myocardial fibrosis that they mediate. In addition, ACEI can activate the ACE2 - Ang 1–9 signaling pathway and block the degradation of Ang 1–7 to alleviate myocardial fibrosis. A study showed that the first-generation ACEI drug captopril significantly inhibited the expression of inflammatory factors such as CXCL8 and IL6, ameliorating the inflammatory response and apoptosis associated with SARS-CoV-2, which may help guide the clinical management of COVID-19-associated cardiac fibers (Huang X. et al., 2023). Treatment with the second-generation ACEI analog enalapril in a rat model reduced ROS, p38MAPK, and TGF-β (Zhou et al., 2020) protein expression, blocked Ang II-induced cardiac fibroblast proliferation, and attenuated myocardial fibrosis (Yu et al., 2012). Another second-generation drug, benazepril, attenuates myocardial fibrosis by modulating TGF-β/Smad signaling proteins (Meng et al., 2009). In conjunction with the above, it is clear that the expression of these proteins is closely associated with the formation of COVID-19-associated myocardial fibrosis. The latest generation of ACEIs has also been shown to be significant in reducing myocardial fibrosis. Lisinopril has been shown to decrease collagen deposition, accompanied by reduced left ventricular stiffness and improved left ventricular diastolic function in patients with or without heart failure (Chang et al., 2016; Brilla et al., 2000). Perindopril reduces cardiac fibrosis by inhibiting the AngII/AT1R pathway, an important mechanism by which SARS-CoV-2 causes fibrosis (Liang H. et al., 2023). The more widely used ramipril inhibits fibrosis by blocking upregulation of collagen I and III transcription (Nagasawa et al., 1995). However, the RAMIC study found that ramipril did not significantly improve or worsen clinical outcomes in COVID-19 patients compared to placebo (Ajmera et al., 2021; Huang D. Q. et al., 2023). These findings offer new insights for optimizing clinical drug use in the management of COVID-19. With the development of the ACEI class of drugs, the first generation of drugs has seen a gradual decrease in side effects, an increase in metabolic pathways, and improvements in half-life and economics. Unfortunately, clinical studies on ACEI analogs for the treatment of COVID-19-associated myocardial fibrosis are still lacking, which provides a new research direction for the future.

#### 4.2.2 Angiotensin II type 1 receptor blockers

A meta-analysis that included 30 studies showed that the application of ARB analogs significantly reduced the risk of severe disease in patients with COVID-19 (Xie et al., 2022). On one hand, as an ARB, telmisartan rapidly and continuously reduces the level of plasma C-reactive protein in hospitalized COVID-19 patients and telmisartan treatment can shorten the hospitalization time of sufferers (Rothlin et al., 2021). What’s more, telmisartan may have an anti-inflammatory effect that reduces the probability of ICU admission, the probability of mechanical ventilation, and the mortality of COVID-19 inpatients (Duarte et al., 2021). On the other hand, telmisartan inhibits hyperglycemia-induced cardiac fibrosis through the PPARδ/STAT3 pathway (Chang et al., 2016). RAAS inhibitors not only diminish Ang II and aldosterone directly but they have also been proven to indirectly boost ACE2 activity (Ferrario et al., 2005) and Ang 1–7 expression, alleviating cardiac fibrosis. Paradoxically, however, RASS inhibitors cause an augmentation of the viral entry receptor ACE2, which may give rise to the toxicity of SARS-CoV-2 in the heart and ultimately accelerate the development of myocardial fibrosis. Even so, a recent randomized controlled study concluded that ACEIs and ARBs have no adverse outcome on the clinical prognosis of COVID-19 individuals with hypertension (Wang W. et al., 2021). Their applications may even be beneficial and protective, but larger-scale studies are needed to confirm these effects in the future. In addition, in a study estimating the therapeutic efficacy of Candesartan in SARS-CoV-2 infected cell lines, it was found that viral infection was positively correlated with the upregulation of pro-fibrotic genes but was normalized by Candesartan. Therefore, it can be hypothesized that candesartan can be used to treat COVID-19-associated myocardial fibrosis (Swiderski et al., 2023; Elkahloun and Saavedra, 2020). Similarly, Candesartan was able to shorten the length of hospitalization in clinical trials (Lukito et al., 2021).

#### 4.2.3 Mineralocorticoid receptor antagonists

MRAs, which can be classified as steroidal and nonsteroidal, reduce the severity of SARS-CoV-2 infection by inhibiting the deleterious effects of aldosterone, thereby attenuating myocardial fibrosis (Kim et al., 2022). The most common of the steroidal MRAs are spironolactone and eplerenone. Spironolactone can improve left ventricular diastolic dysfunction and reduce ventricular stiffness, which is related to the regression of myocardial fibrosis (Izawa et al., 2005). It was investigated that in Coxsackievirus B3m (CVB3)-induced viral myocarditis in mice, early intervention with eplerenone modulated the acute host defense response and prevented the progression of CVB3-induced myocarditis. Eplerenone treatment resulted in a marked reduction in collagen content in the left ventricle compared to untreated CVB3-infected mice, leading to early and permanent improvements in left ventricular size and function. The outcomes of this research advocated the premature use of eplerenone for the treatment of viral myocarditis, even before the onset of inflammation-induced myocardial dysfunction (Tschope et al., 2020), this provides insight into the treatment of COVID-19-associated myocardial fibrosis. Furthermore, several studies have suggested that spironolactone and eplerenone may be prospective options for attenuating endothelial inflammation in SARS-CoV-2 infection (Jover et al., 2021), which also contributes to myocardial fibrosis in COVID-19. Both drugs have also demonstrated their role in treating COVID-19 in the clinic. A prospective randomized clinical trial discovered that compared with the matched group, the clinical score of COVID-19 individuals treated with spironolactone was notably lower on the fifth day after admission, and the mortality rate was also lower (Abbasi et al., 2022). In another clinical trial compared with the control group, the group treated with bromhexine and spironolactone had a shorter hospital stay and faster clearance of SARS-CoV-2 (Mareev et al., 2020). However, as research has progressed, the effectiveness of these two drugs in treating COVID-19 has been questioned. The results of a meta-analysis showed no significant association between mortality in SARS-CoV-2 infected individuals and MRA treatment, suggesting that there is no clear benefit of MRA treatment for SARS-CoV-2 infection (Kim et al., 2022), which would require larger, longer randomized controlled trials to elucidate this relationship. Meanwhile, although there have been many studies demonstrating the antifibrotic effects of spironolactone and eplerenone in different tissues, these studies have been limited to rodent models and there is no direct evidence of the beneficial effects of steroidal MRAs on fibrosis after viral infections (He et al., 2013; Barut et al., 2016; Zhou et al., 2016; Kotfis et al., 2021). Furthermore, the use of spironolactone and eplerenone both cause an increase in hyperkalemia, while spironolactone can also cause breast pain and gynecomastia (Agarwal et al., 2021; Pitt et al., 1999). These side effects can be ameliorated by a class of highly selective, nonsteroidal MRAs such as finerenone and esaxerenone (Agarwal et al., 2021; Kolkhof and Bärfacker, 2017).

Nonsteroidal MRAs had a stronger antagonistic effect on MR, were able to achieve complete MR blockade at lower doses, and both reduced myocardial fibrosis (Arhancet et al., 2010; Rahman et al., 2021). It has been shown that finerenone reduces the expression of pro-fibrotic markers and improves macrophage invasion in a short-term isoprenaline-induced model of cardiac fibrosis in mice, and has a more potent anti-fibrotic effect than the steroid MRA (Grune et al., 2018). In rats fed a high-salt diet, esaxerenone significantly reduced the expression of cardiac fibrosis markers such as TGF-*β*, type I and type III collagen (Rahman et al., 2021). However, direct evidence for the treatment of COVID-19-associated myocardial fibrosis with nonsteroidal anti-inflammatory drug MRAs is still lacking. Therefore, in the future, we may consider focusing more on non-steroidal MRAs for the treatment of COVID-19-associated myocardial fibrosis. In conclusion, there is an arduous way to go to study the mechanism of antifibrotic therapy with MRAs.

The above theoretical analysis will be the necessary first step to clarify the role of RAAS inhibitors in COVID-19-associated myocardial fibrosis. Further clinical studies are warranted to observe and describe the efficacy of RAAS inhibitors on COVID-19-related myocardial fibrosis.

### 4.3 Immunomodulators

#### 4.3.1 Tocilizumab

IL-6, a vital pro-fibrotic inflammatory factor (Abbasi et al., 2022), was significantly elevated in the inflammatory factor storm of COVID-19 patients (Coomes and Haghbayan, 2020). Tocilizumab is a humanized monoclonal antibody against the IL-6 receptor that has been broadly prescribed for the treatment of patients with COVID-19 (Sheppard et al., 2017; Lan et al., 2020). Meta-analysis illustrated that tocilizumab treatment displayed promising results in terms of decreasing 28-day mortality and progression to mechanical ventilation in patients with moderate to severe COVID-19. And its application did not pose a burden of serious adverse events (Yu et al., 2022). As mentioned above, neutering IL-6 was shown to reduce cardiac fibroblast activation in mice, so we propose that tocilizumab may also have a bearing on combating myocardial fibrosis caused by COVID-19, but clinical epidemiological evidence is not currently available. Nonetheless, studies have confirmed the potency of tocilizumab in treating myocardial fibrosis arising from systemic sclerosis (Ishizaki et al., 2021). Thereby, tocilizumab may be a dormant drug for treating COVID-19-associated myocardial fibrosis.

#### 4.3.2 Anakinra

Likewise, anakinra is a high molecular weight recombinant of IL-1 receptor antagonist. In a clinical trial, the treatment of anakinra plus methylprednisolone cut down the mortality of patients with severe COVID-19 (Bozzi et al., 2021). Antagonists of IL-1βRs could prevent IL-1β from promoting fibrosis by reducing the binding of IL-1βR to IL-1 Anakinra was shown to alleviate myocardial fibrosis triggered by radiation in mice (Mezzaroma et al., 2015). Thus there may be a bonus gain of anti-myocardial fibrosis when treating COVID-19 with anakinra.

### 4.4 Other drugs

Colchicine, extracted from *Colchicum autumnale*, has anti-inflammatory as well as antiviral properties. Colchicine treatment has been reported to be efficacious in case reports of patients with COVID-19 infection (Hossen et al., 2020). Clinical trials proved that the application of colchicine could shorten the clinical deterioration time and hospitalization time of COVID-19 individuals, and reduce oxygen therapy (Lopes et al., 2021; Deftereos et al., 2020). It was demonstrated that colchicine dramatically restrained macrophage proliferation and migration. In a mouse model of myocardial infarction, colchicine reliably attenuated cardiac inflammation and curbed cardiomyocyte apoptosis and fibrosis. Consequently, cardiac function and structure are improved and mice survival is increased without causing serious systemic toxicity (Chen Y. et al., 2020). Anti-myocardial fibrosis may be a windfall for colchicine treatment of COVID-19.

The synthetic glucose analog 2-DG is a glycolysis inhibitor. A multicenter, randomized phase II clinical, experimental data demonstrated that application of 2-DG reduces the need for supplemental oxygen, and thus 2-DG is expected to be used as adjunctive therapy to standard of care (Bhatt et al., 2022b). What’s more, SARS-CoV-2 infection leads to impaired mitochondrial metabolism and enhanced glycolytic pathways in host cells, and 2-DG can inhibit glycolysis and stimulate respiration (Bhatt et al., 2022a; Codo et al., 2020; Bojkova et al., 2020a; Aiestaran-Zelaia et al., 2022). It has been found that the inhibition of glycolytic pathway by 2-DG treatment attenuated the Nsp6-induced cardiac phenotype in Drosophila and mice, suggesting that glycolysis is a potential pharmacological target for treating COVID-19-related cardiac injury (Zhu et al., 2022). In addition, in a prospective cohort study, the use of the propensity score matching method yielded that Nirmatrelvir-ritonavir was effective in reducing myocardial injury and long-term adverse cardiovascular outcomes in COVID-19 hospitalized patients (Gu et al., 2024).

## 5 Future directions in the post-COVID-19 era

At present, there have been tremendous advances in the treatment of COVID-19 (Alexander et al., 2023), but comparatively little attention has been paid to COVID-19-associated myocardial fibrosis. Simultaneously, both its mechanism and treatment have sparsely been investigated. Few of the above drugs have been studied in clinical trials to demonstrate their efficacy in COVID-19-associated myocardial fibrosis. This provides novel research directions for the future.

COVID-19 can deliver not only short-term damage, but with the widespread prevalence of COVID-19, the long-term impairments known as PPCS have acquired growing attention. A two-way cohort study found that 68% of COVID-19 survivors developed at least one PPCS-related symptom 6 months after acute infection, most commonly fatigue, sleep difficulties, muscle weakness, and in more severe cases chest imaging abnormalities and pulmonary diffusion dysfunction (Huang C. et al., 2023). Therefore, PPCS is responsible for prolonging hospital stays and raising overall mortality from disease. Early prevention and detection of PPCS is particularly valuable given the devastation that higher healthcare utilization and lost productivity can have on a shrinking economy. COVID-19-related myocardial fibrosis is likely to be an essential part of PPCS. In a retrospective study that included 26 patients recovering from COVID-19, eight cardiac magnetic resonance (CMR) demonstrated late gadolinium enhancement suggestive of edema and fibrosis (Huang L. et al., 2020). A 45-year-old woman with no history of myocarditis presented with cardiac palpitations and non-typical chest pain 3 months after COVID-19 infection, and CMR showed diffuse interstitial fibrosis (Jagia et al., 2020). Thus, patients recovering from COVID-19 may have persistent cardiac involvement, which provides a novel idea as to whether myocardial fibrosis examination should be included as a routine examination item during the follow-up of patients with COVID-19 after discharge.

Extensive research has demonstrated that patients with heart disease and COVID-19 are at high risk for hospitalization complications and long-term mortality (Sokolski et al., 2023). The influenza pandemic experience could lead to a deeper understanding of patients with myocardial fibrosis and optimization of clinical services in the more distant future, beyond the pandemic. During the COVID-19 pandemic, telemedicine services developed rapidly, encouraged by professional associations, in response to patient demand and government decisions (Sammour et al., 2021). Despite the end of the COVID-19 pandemic, numerous telemedicine applications are likely to have a significant role in recovering patients with cardiovascular disease. Integrating telemedicine systems and infrastructure that could acquire real-time data access with standard clinical care could refine the management of heart disease in the post-COVID-19 era, such as remote monitoring of cardiac implantable devices and telerehabilitation (Bellicini et al., 2023). Moreover, artificial intelligence (AI) telemedicine could be an indispensable part of tailored medicine to achieve the effectiveness and quality of care in patients with myocardial fibrosis. In the era of the persistent emergence of drug-resistant pathogens, COVID-19 has highlighted the urgent demand for precision medicine which requires a new knowledge network to innovate disease taxonomy for more precise diagnosis, therapy, and disease prevention (Cao Q. et al., 2023).

## 6 Conclusion

Given the global burden of COVID-19 and the virulence of SARS-CoV-2, radiological and pathological evidence of COVID-19-related myocardial fibrosis is increasingly cropping up. However, there is still a lack of comprehensive insights into its molecular mechanism, and this review innovatively and systematically summarizes the potential mechanisms of COVID-19-associated myocardial fibrosis at the molecular and cellular levels. SARS-CoV-2 can activate cardiac fibroblasts to lead to myocardial fibrosis through the mediation of RAAS, TGF-β1, and so on. Similarly, immune cells, endothelial cells, pericytes, and cardiomyocytes are also involved in the deleterious process. Under the joint participation of various cells and fibrogenic substances, SARS-CoV-2 expedites the deposition and cross-linking of collagen, accounting for the occurrence of myocardial fibrosis. Nevertheless, studies on the potential mechanisms of COVID-19-associated myocardial fibrosis remain unclear, and further investigations are needed to gain a deeper understanding of COVID-19-associated myocardial fibrosis and to lay the foundation for subsequent clinically targeted therapies. At present, there is no proven treatment option for COVID-19 individuals who develop myocardial fibrosis. It is urgent for clinicians and researchers, as well as the healthcare system, to embrace this challenge to minimize disability, maximize the quality of life, and prevent another global health disaster. In this paper, we attempt to find out the drugs with anti-fibrosis effects from the existing COVID-19 treatment strategies such as remdesivir, lisinopril, and telmisartan, which offer viable options for the clinical treatment of COVID-19-related myocardial fibrosis. Unfortunately, these drugs have not been fully proven in the clinic and further clinical studies are needed to assess their efficacy and safety against myocardial fibrosis. In addition to short-term damage, the long-term sequelae caused by COVID-19 should not be underestimated. Myocardial fibrosis may be an important part of PPCS. We suggest that relevant examinations should be added in the follow-up of COVID-19 patients after discharge to find myocardial fibrosis in time and minimize the damage. Combining telemedicine and artificial intelligence should be implemented to achieve progress in healthcare quality.
